# Cancer Radiotherapy: Understanding the Price of Tumor Eradication

**DOI:** 10.3389/fcell.2020.00261

**Published:** 2020-04-24

**Authors:** Olga A. Martin, Roger F. Martin

**Affiliations:** ^1^Sir Peter MacCallum Department of Oncology, The University of Melbourne, Melbourne, VIC, Australia; ^2^School of Chemistry, The University of Melbourne, Melbourne, VIC, Australia

**Keywords:** cancer radiotherapy, quality of life, normal tissue toxicity, aging, systemic effects, DNA damage, DNA repair, radioprotectors

Cancer radiotherapy (RT) is involved in the treatment of more than a half of all cancer patients, because it is highly effective; 40% of cancer cures can be attributed to RT (Baskar et al., 2012). Moreover, the efficacy of RT is steadily improving, largely due to the striking progress in technology, aimed at maximizing the radiation dose to the tumor and minimizing the dose to normal tissues. This continual improvement contributes to the increasing numbers of cancer survivors. In Australia, the 5-years relative survival from all cancers (excluding skin cancer) increased from 48% in 1984–1988 to 68% in 2009–2013^1^. In 2012, 410,530 ex-cancer patients were alive 5 years after treatment; 1.8% of the population. In the USA, there are now 14M cancer survivors; ~4% of the population (Travis, 2006; Travis et al., 2013). Accordingly, increasing attention is now directed to the quality of life (QoL) of cancer survivors, particularly to treatment-related toxicities (Stone et al., 2003; De Ruysscher et al., 2019), as highlighted in a recent report from the National Cancer Research Institute in the UK^2^.

Normal tissue toxicity from RT can be attributed to three different etiologies. The most obvious of these can be defined as “targeted”, due to relatively high radiation doses to normal tissues in the vicinity of a tumor. Ironically, the technological improvements in dose delivery that have diminished this problem, have contributed to the second category of normal tissue toxicity. Modern RT techniques (e.g., Intensity-Modulated RT, IMRT) use multiple moving beams that sculpt a volume of high dose encompassing the tumor, so quite large volumes of normal tissues are ‘bathed' in low doses, within and between beams (Kry et al., 2005; Harrison, 2017). This category also includes scattered radiation that spreads out in different directions from each radiation beam. The third category can be considered as “systemic,” reflecting the radiation-induced abscopal (“out-of-field”) effect (RIAE). This is attributed to the localized stress in the irradiated volume, that triggers a systemic biological response that is propagated to sites distant from the irradiated volume, and is largely mediated by the immune system (Reynders et al., 2015; Siva et al., 2015). In a sense, the RIAE can be considered as the systemic counterpart of the cellular radiation-induced bystander effect (RIBE), although the historical understanding of the phenomena was quite different. The recognition of the RIBE (Nagasawa and Little, 1992; Prise and O'Sullivan, 2009) is much more recent, compared to early observations the RIAE by radiation oncologists, that manifest both as out-of-field tumor responses and out-of-field RT-associated toxicities (Mole, 1953; Siva et al., 2015).

The best-known RT-induced normal tissue toxicities are targeted effects (tissue responses in the higher dose volume), the subject of many classical and contemporary radiobiological studies (Stewart and Dorr, 2009). They can be acute (appear within weeks of irradiation), late (months to years after RT), or both. For targeted effects, there is a wide spectrum of individual radiosensitivity (RS) manifested as normal tissue toxicity (Barnett et al., 2009). Low dose- and RIAE-generated “silent” toxicities, e.g., chronic inflammatory responses and mutagenesis in radiosensitive tissues, can also lead to long-term tissue dysfunction, even for future generations (Dubrova, 2003). Just as it is well-established for targeted effects, one can expect that there will be a spectrum of individual RS for low dose and systemic effects.

Epidemiological findings in long-term cancer survivors treated with RT indicate the increased incidence of degenerative pathological conditions normally associated with aging, or age-related diseases (e.g., cardio- and cerebrovascular disorders, neurodegeneration including dementia, hormonal disturbances, cataracts, bone marrow insufficiency, immune system dysfunction, second cancers, and overall life shortening) (Cupit-Link et al., 2017). Evidence is accumulating for similar consequences of low dose IR exposure (Majer et al., 2014; Harrison, 2017). Therefore, aging may be the common link between the diverse late morbidities and RT. By amplifying the mechanisms that are responsible for cellular aging (Sabatier et al., 1995; Dubrova et al., 2002; Dubrova, 2003; Miller et al., 2008; Paulino et al., 2010; Azzam et al., 2012; Sabatino et al., 2012; Merrifield and Kovalchuk, 2013; Ungvari et al., 2013; Sprung et al., 2015; Shimura et al., 2016; Yin et al., 2018), RT may induce a premature aging manifested as an accelerated onset of chronic degenerative disorders in some patients. Figure 1 highlights the similarities between the response to IR and aging, but there are some differences, e.g., differences in the spectra and severity of DNA lesions (Nikitaki et al., 2015). Premature cellular senescence (Nakamura et al., 2008) is also an important common feature of the two pathologies. It is important to note that the idea of RT-induced accelerated aging is not a new one (Richardson, 2009), but given the growing aging population, it has increasingly important consequences for the cost of community health care. Moreover, the availability of improved biomarkers provides a means of monitoring both the need for intervention, and the efficacy of proposed interventions.

**Figure 1:**
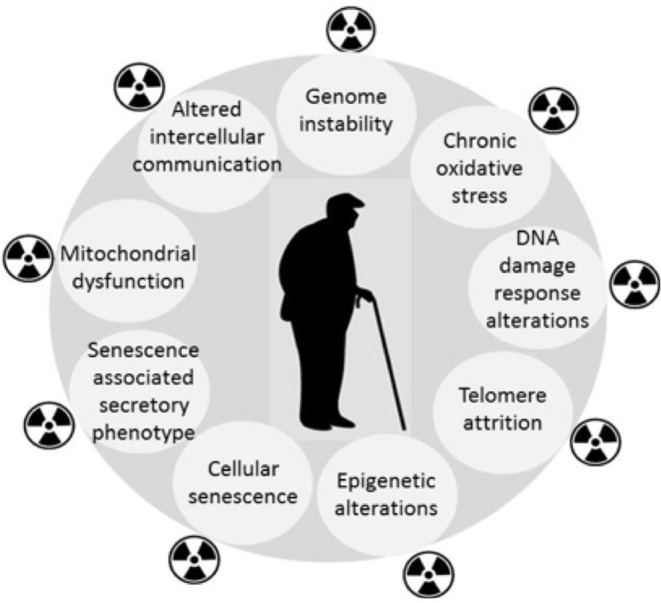
RT amplifies processes reminiscent the biological hallmarks of aging.

Therefore, to fully understand the role of RT in accelerating the aging process, research aimed at the following objectives is required: (1) Development of a “signature” profile of systemic markers to identify RT cancer patients susceptible to development of premature aging; (2) Improving mechanistic understanding of systemic propagation of genotoxic events and the associated aging phenotype following local exposure to IR; (3) Development of strategies for prevention, protection and mitigation of RT-related systemic genotoxic events.

## Identification of a Signature of Systemic Markers for Premature Aging in RT Cancer Patients

The kinetics of aging biomarkers could be monitored in blood of RT-treated cancer patients and compared with the pre-treatment values. Suitable patients, scheduled for treatment with RT, would be <50–60 years of age, and without evidence of non-cancer morbidities at the time of work-up. Examples of a suitable cohort would be breast or head & neck cancer patients, with an anticipated 5-years survival >90%.

Studies indicate that accumulated unrepaired systemic DNA damage underlies RT-induced pathologies (De Ruysscher et al., 2019). The DNA damage response (DDR) varies in young, mature and old mice, slow down with age, making old mice the most vulnerable to radiation effects (Kovalchuk et al., 2014). DDR declines in senescent cells and during normal and premature human aging (Sedelnikova et al., 2008), and individual RS continuously rises with age (Schuster et al., 2018). Novel functional assays of radiation-induced DNA damage recognition and repair efficiency in *ex-vivo* irradiated primary human fibroblasts and peripheral blood mononuclear cells (PBMC) have been recently developed. The tests are based on post-irradiation formation of nuclear repair foci at the sites of DNA double-strand breaks (DSBs) for two DNA damage markers, phosphorylated ATM kinase (“ATM nucleo-shuttling”) (Bodgi and Foray, 2016; Pereira et al., 2018) and histone H2AX (γ-H2AX) (Martin et al., 2013; Lobachevsky et al., 2015a, 2016). Both assays efficiently separated radiosensitive individuals with impaired DDR from those with normal RS. In our retrospective study, ex-RT patients who had documented to have severe RT-induced toxicity (and matched controls who responded normally) were recalled for blood sampling. A novel statistical algorithm was developed by Lobachevsky et al. (2016), based on non-linear regression analysis of the kinetics of repair of γ-H2AX foci, following *ex-vivo* irradiation of the PBMC. Subsequently the same dataset was analyzed by Bayesian modeling (Herschtal et al., 2018). Both methods of analysis distinguished the radiosensitive patients from controls, but the Bayesian statistics also outlined the importance of assessment of both the initial radiation-induced DNA damage and DNA damage repair. In a later study, the *ex-vivo* γ-H2AX response was assayed in PBMC collected before and during RT, and this showed that RT itself can affect individual RS, as reflected by changes in DSB repair efficiency in PBMC (Yin et al., 2018), adding a further dimension to the challenge of implementation.

Also, the number of endogenous γ-H2AX foci per cell in PBMC of normal individuals increases with age in a linear fashion (Sedelnikova et al., 2008; Schurman et al., 2012). The outliers identified in the linear regression analysis (Schurman et al., 2012) included elevated γ-H2AX foci/cell in patients with clinical morbidities. Interestingly, DDR has been linked with the immune response for normal and tumor tissues, as evidenced by cumulative bioinformatics studies (Georgakilas et al., 2015).

Therefore, the numbers of γ-H2AX foci/cell and efficiency of DDR in PBMC could provide a basis for identification of RT patients susceptible to RT-induced premature aging. However, it is more likely that a combination of markers will be required to constitute an effective “signature” to identify patients requiring added attention. Candidates for such auxiliary biomarkers include those reflecting immune and epigenetic alterations, increased immune cell senescence, oxidative stress, and mitochondrial dysfunction.

## Improving the Mechanistic Understanding of Systemic Propagation of Genotoxic Events and the Associated Aging Phenotype Following Local Exposure to IR

Conventional RT triggers systemic biological effects in animal models (Koturbash et al., 2006, 2008; Mancuso et al., 2008), but due to significant scatter, RIAEs are difficult to interpret. The scatter problem associated with conventional radiation sources is much reduced with Synchrotron radiation, providing a useful tool to study RIAE. The defined geometry and coherence of the synchrotron beam delivers IR to small volumes with lower scatter, and the high dose rate (up to >1,000 Gy/sec) minimizes motion artifacts, but also introduces the “FLASH” effect (Durante et al., 2018). Ventura et al. (2017) reported that that various synchrotron settings (IR dose, volume, beam modality) trigger similar systemic effects in normal mouse tissues of wild-type C57BL/6 mice. Depending on the level of scatter radiation (Lobachevsky et al., 2015b), these effects were attributed to either true abscopal signaling, or to direct low-dose scatter radiation. RIAE was abrogated in mice with immune deficiencies, e.g., in mice with non-functional macrophages (Lobachevsky et al., 2019). Possible extensions of these studies using synchrotron irradiation include comparison of targeted and out-of-field effects of IR in young and old mice of wild-type and immune-deficient strains, as well as verification of salient features using a model with conventional radiation beams. The objective would be to understand the pathways by which IR modulates the aging processes in various organs crucial for the development of IR-related late pathologies (e.g., spleen, bone marrow, heart, vasculature, gonads, brain). These experimental models could also be used to evaluate potential therapeutic targets that emerge from the clinical studies described in the previous section.

## Development of Strategies for Prevention, Protection and Mitigation of RT-Related Systemic Genotoxic Events

Targeted radiation effects and RIAE are both associated with elevated DNA damage and genome instability mediated by reactive oxygen species (ROS), so it is critical that any strategy aimed at reducing systemic genotoxic events does not compromise the cancer therapy, mediated by targeted radiation effects. Similarly, whilst normal tissue toxicities associated RIAE are mediated by the immune response, the tumor response to RT also involves the immune response (Haikerwal et al., 2015; Xing et al., 2019). However, kinetic studies on the impact with immunomodulators on the response of targeted tumors, out-of-field metastases and RIAE in normal tissues may reveal differences in response kinetics enabling selective suppression of RIAE in normal tissues. Such kinetic differences enabled scatter effects to be distinguished from the systemic RIAE (Ventura et al., 2017).

Our mouse studies revealed that molecules that block cytokines/cytokine receptors and macrophages can be expected to mitigate abscopal genotoxic events in normal tissues (Ventura et al., 2017; Lobachevsky et al., 2019). Our extensive review of potential strategies for prevention of RT-induced second cancers (Martin et al., 2016) illustrates the range of approaches that can be considered for all toxicities mediated by RIAE. A review on radiation-induced cardiotoxicity (Stewart et al., 2013), which noted the role of systemic effects, also discussed strategies for prevention. Another report extensively reviewed strategies for amelioration of radiation effects on the eye (Kleiman et al., 2017), some of which could be applicable to RT. In this context, a relatively new family of radioprotectors developed by one of the authors (RFM) is of interest. The first example, methylproamine (Martin et al., 2004), is a potent radioprotector *in vitro*; a dose modification factor of 2.0 at a concentration of 10 uM (Lobachevsky et al., 2011) and improved analogs, including *in vivo* activity, have been reported in the patent literature (Martin et al., 2011). Such radioprotectors have the potential to take advantage of the slow kinetics observed for the RIAE, illustrated by the report of the delayed appearance of DNA damage in eyebrow hair follicles after RT of lung cancer patients; 24 h after the first fraction (Siva et al., 2015). A delayed DDR is well-established for the cellular RIBE (Sedelnikova et al., 2007). Interestingly, methylproamine protects bystander cells in the *in vitro* RIBE setting, e.g., if present with recipient cells at the time of transfer of media irradiated cells (Burdak-Rothkamm et al., 2015). By contrast, in the context of targeted radiation effects, methylproamine must be present before and during irradiation to endow radioprotection of cultured cells (Lobachevsky et al., 2011), consistent with the mechanism (DNA-binding antioxidant) of radioprotection (Martin and Anderson, 1998). Thus, one can envisage an RT scenario in which such radioprotectors could be administered after irradiation, and thus not compromise response of the tumor, but nevertheless mitigate the subsequent RIAE mediated toxicity in normal tissues. Whilst scheduling that avoids the possibility of compromising the tumor response might be challenging in the setting of conventional fractionation, this would be less problematical for hypofractionation modalities.

## Conclusions

RT has an established role in cancer therapy and is unlikely to be superseded in the foreseeable future. In addition to pursuing better treatments, it is time now for more focus on the QoL of cancer RT survivors. The priorities include the need to understand the biological basis of treatment side-effects and their management, and, in particular, the mechanisms responsible for RT-induced aging phenotype and associated pathologies. This new knowledge is expected to enable development of systemic markers to identify patients most susceptible to accelerated aging, and the early stages of that process, as well as novel interventions for prevention and mitigation. Thus, the overall objective is early diagnosis, monitoring and management of RT-related morbidities, and identification of those cancer patients at most risk of these morbidities so their treatments can be modified accordingly.

## Author Contributions

OM and RM contributed ideas and wrote the manuscript.

## Conflict of Interest

RM has a commercial interest associated with his intellectual property on DNA-binding radioprotectors. The remaining author declares that the research was conducted in the absence of any commercial or financial relationships that could be construed as a potential conflict of interest.

## Footnotes

^1^Cancer in Australia Australian Association of Cancer Registries, 2017.

^2^https://www.ncri.org.uk/lwbc/
